# Chronic Gonorrhœa

**Published:** 1874-11

**Authors:** 


CHRONIC GONORRHŒA.—The case was one that had resisted every form of treatment that had been adopted, which had been numerous. Injections of earth, rendered sufficiently liquid with water that it can be thrown through a syringe, were employed, and the patient was nearly well. The remedy had been used only a short time. Acute gonorrhœa is treated in the same manner.—*Penn. Hosp. Reports.*

